# Tea bags as hidden source of micro- and nanoplastics: a systematic review for food safety

**DOI:** 10.1016/j.crfs.2026.101425

**Published:** 2026-04-30

**Authors:** Mohammad Ehsan Sanei, Amirhossein Abedini, Arezou Khezerlou, Gholamreza Jahed-Khaniki, Nabi Shariatifar, Ebrahim Molaee-Aghaee, Mahmood Alizadeh Sani

**Affiliations:** a Department of Environmental Health Engineering, School of Public Health, Tehran University of Medical Sciences, Tehran, Iran; b Student Research Committee, Department of Food Science and Technology, Faculty of Nutrition Science and Food Technology, National Nutrition and Food Technology Research Institute, Shahid Beheshti University of Medical Sciences, Tehran, Iran; c Department of Food Safety and Hygiene, School of Health, Gonabad University of Medical Sciences, Gonabad, Iran; d Department of Food Safety and Hygiene, School of Public Health, Tehran University of Medical Sciences, Tehran, Iran; e Department of Food Science and Technology, School of Nutritional Sciences and Dietetics, Tehran University of Medical Sciences, Tehran, Iran

**Keywords:** Micro/nanoplastics, Tea bag, Food safety, Release, Systematic review

## Abstract

**Background:**

The release of micro- and nanoplastics (MNPs) from tea bags during brewing is a significant, recently identified source of potential human exposure. This systematic review collected global research published up to the end of 2025, indexed in PubMed, ScienceDirect, Web of Science, and Scopus, adhering to Preferred Reporting Items for Systematic Reviews and Meta-Analyses (PRISMA) guidelines. Study objective was to systematically detect, quantify, and characterize MNPs released from various tea bag materials. The review focused exclusively on studies that directly measured MNPs release under brewing conditions.

**Methodology:**

A systematic literature search was conducted through specified databases. Inclusion criteria focused on studies quantifying MNPs release (by count or mass) from tea bags during brewing, irrespective of water temperature or time, but studies relying on in-vitro or in-vivo models, review/conference/retracted papers, or books/chapters were excluded. Data extraction included MNPs type, quantity, size range, tea bag material, brewing conditions, and detection methods.

**Main findings:**

Our systematic search identified 19 studies meeting the inclusion criteria. A significant finding is the substantial release of MNPs, often in billions of particles per bag, from plastic-containing tea bags (e.g., non-woven polypropylene, nylon) brewed in hot water (typically ≥95 °C). Released particles predominantly ranged from nano-to micrometer sizes. Release quantities were found to be influenced by tea bag material, water temperature, brewing duration, and mechanical agitation. While some materials like paper or woven nylon showed lower release rates, even composite or supposedly “eco-friendly” bags contributed measurable MNPs release.

**Limitations and gaps:**

Despite the direct evidence of MNPs release, some research gaps persist. Crucially, standardized methodologies for detection and quantification of MNPs, and human health risk assessment are lacking, obstructing comparability across studies. Furthermore, while our review focused on MNPs release, the in-vivo or in-vitro evidence regarding the biological implications remains largely outside the scope of this specific systematic review's direct findings, necessitating separate, targeted investigations. Current global exposure estimates are preliminary due to these methodological and data limitations.

**Conclusion:**

This systematic review confirms that tea bags, particularly those containing plastics, are a notable source of MNPs release into beverages and environment. While considerable quantities of MNPs are released, future systematic efforts should highlight developing and implementing standardized detection methodologies to improve the reliability of findings, alongside focused investigations into the biological and toxicological effects of MNPs and human health risk assessment. Furthermore, exploring and promoting innovative solutions, such as the use of biodegradable materials with minimal or without MNPs releasing and alternative brewing technologies, is crucial for mitigating MNPs contamination from tea consumption.

## Highlights

• Billions of MNPs released from tea bag (polypropylene, nylon) in hot water (≥95 °C).
• Nano-to micrometer sizes dominate MNP release from tea bags, varying by material.
• Material, temperature, brewing duration, agitation influence MNPs release content.
• Paper/woven nylon bags showed lower release rates than plastic ones.
• Urgent need for regulations, biomaterials, and awareness to reduce MNPs exposure.

## Graphical abstract

**Figure:**
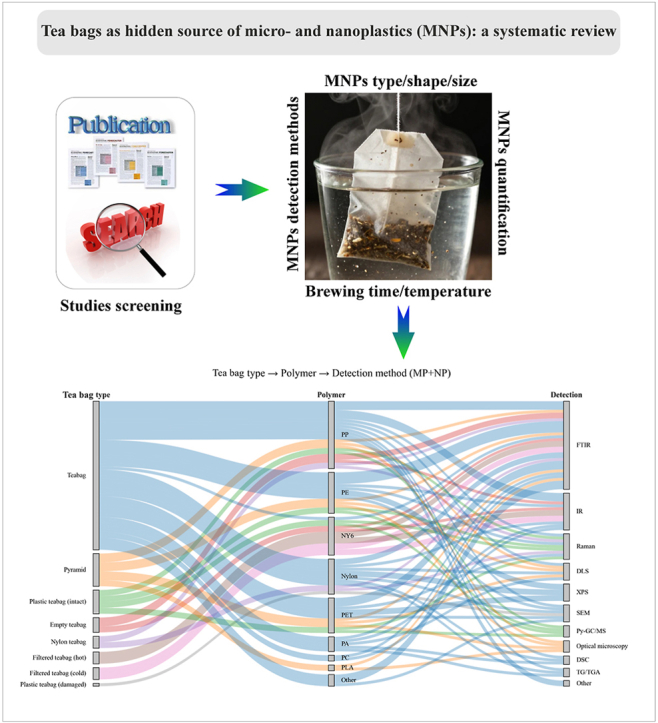

## 1 Introduction

Micro (1 μm-5mm), and nanoplastics (1nm-1μm) (MNPs) are increasingly recognized as a pervasive environmental contaminant with potential human health implications, entering the food chain through various dietary sources, including beverages ((WHO) 2019, Fard et al., 2025; Lamoree et al., 2025). Accordingly, the infiltration of MNPs into the food chain through contaminated beverages and foods such as bottled water, tea, table salt, seafood, honey, and dairy products has become a serious public health concern that demands urgent investigation (Iñiguez et al., 2017; Smith et al., 2018; Diaz-Basantes et al., 2020; Gambino et al., 2023; Kashfi et al., 2023).

Tea, one of the world's most consumed beverages, represents a significant dietary pathway for MNPs exposure, especially given the widespread use of tea bags for convenience (Boonerjee et al., 2025; Kumar and Verma, 2025). According to global estimates, tea consumption in 2020 was about 6.3 million metric tons and is expected to increase to about 7.4 million metric tons by 2025, indicating a growing international demand (Kumar et al., 2023). Similarly, the global tea bags market is set to grow from USD 7.69 billion in 2025 to USD 14.59 billion by 2035, adding USD 6.90 billion in new revenue and advancing at a CAGR of 6.6%. In modern consumption habits, tea is predominantly prepared using tea bags, primarily due to their convenience, ease of disposal, rapid brewing time, and flexibility in ingredient blending (Bassi et al., 2020); however, this convenience comes with an emerging contamination risk.

Early research indicated that MNPs could be present in tea leaves themselves or introduced via processing water (Li et al. 2022, 2025). However, a growing body of evidence points towards the tea bag material as a distinct and significant source of MNPs contamination (Mei et al., 2022). Studies have revealed that the manufacturing process of tea bags, the materials used (including plastics like polypropylene (PP), nylon, polyethylene terephthalate (PET), and polylactic acid (PLA)), and crucially, the act of brewing in hot water, can lead to the migration of considerable quantities of MNPs into the tea (Hernandez et al., 2019a,b; Mei et al., 2022; Ali et al., 2023). Some studies suggest that even tea bags marketed as “biodegradable” may contain conventional plastics or release MNPs under brewing conditions (Yurtsever, 2021; Courtene-Jones et al., 2024). The MNPs release rate can be considerable, with some studies estimating millions of particles per tea bag (approximately 2.3 million microplastic particles) (Hernandez et al., 2019a,b), while others report lower (∼5800–20,400 particles per bag) but still significant numbers (Busse et al., 2020).

Likewise, major challenges and scientific gaps remain, including the absence of standardized, robust analytical protocols for the extraction, identification, and quantification of MNPs from complex matrices like brewed tea and tea bag materials, the absence of a global database, and insufficient standardized recycling processes. Overall, an integrated approach combining education, waste management, biological solutions, material innovation, and technical advancements are essential to effectively mitigate MNPs pollution (Krishnan, 2023; Xu et al., 2024).

**Fig. 1:**
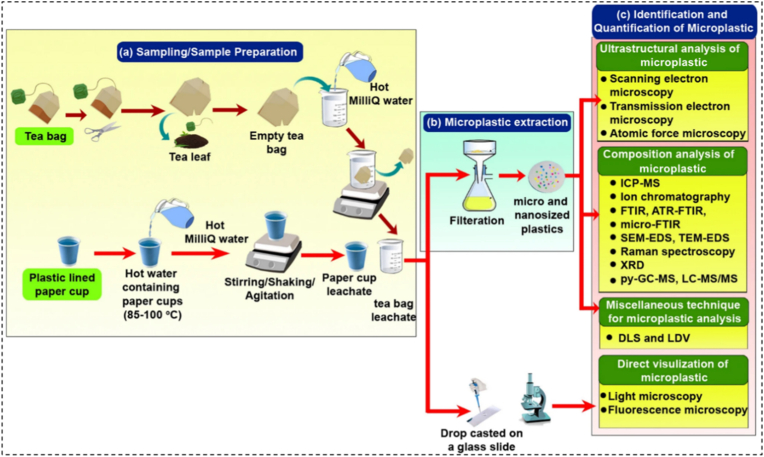
A schematic diagram outlines the process for detecting and identifying microplastics released from paper cups and tea bags, which includes preparing the samples, isolating microplastics from the resulting leachate through vacuum filtration, and then determining the type and amount of microplastics present using different analytical methods, with permission from Ref. (Kumar and Verma, 2025), Open access, Springer publisher. (ICP-MS: Inductively Coupled Plasma - Mass Spectrometry, FTIR: Fourier-Transform Infrared Spectroscopy, ATR-FTIR: Attenuated Total Reflectance - Fourier-Transform Infrared Spectroscopy, SEM-EDS: Scanning Electron Microscopy - Energy-Dispersive X-ray Spectroscopy, TEM-EDS: Transmission Electron Microscopy - Energy-Dispersive X-ray Spectroscopy, XRD: X-ray Diffraction, Py-GC-MS: Pyrolysis - Gas Chromatography - Mass Spectrometry, LC-MS-MS: Liquid Chromatography - Tandem Mass Spectrometry, DLS: Dynamic Light Scattering, LDV: Laser Doppler Velocimetry).

The heterogeneity in findings across existing literature is a major impediment to accurate exposure assessment and risk evaluation (Hernandez et al., 2019; Li et al., 2022; Kashfi et al., 2023; Güzel İzmirli and Gökkaya, 2024; Mondal et al., 2025). This variability is created from numerous factors, including: (i) Differences in sample preparation: Methods for extraction MNPs from tea infusions and residues vary significantly (Fig. 1), (ii**)** Inconsistent MNPs characterization: Techniques used for identifying and quantifying MNPs (e.g., microscopy, spectroscopy) and the size ranges analyzed differ widely, (iii) Influence of brewing conditions: The impact of temperature, brewing time, and water volume on MNPs release is not uniformly studied or reported, (iv) Material diversity: The wide array of materials used in tea bag construction (including novel bioplastics) and potential additives complicates direct comparisons.

Before analytical characterization, appropriate sample pretreatment, such as density separation, filtration, and organic matter digestion, is also critical for reliable quantification; however, overly aggressive treatments may alter polymer morphology and compromise particle integrity (Rani et al., 2023; Sipps et al., 2023). Fig. 1 illustrates the process of detecting and identifying MNPs from paper cups and tea bags, encompassing sample preparation, extraction of MNPs from the leachate via vacuum filtration, and subsequent analysis of their composition and quantity using a range of analytical techniques.

Although some studies have reported the amount of MNPs in tea samples (Fard et al., 2025; Kumar and Verma, 2025) and food packaging materials (Cella et al., 2022), this study specifically explores the migration of MNPs from tea bags, where the type of packaging used in tea bags, shape and size of MNPs, detection methods, as well as the effect of brewing time and temperature, have a significant impact on the amount of these contaminants. Also, this systematic review, aims to critically synthesize current evidence on MNPs release from tea bags, and identify key methodological gaps to guide future research and policy interventions. By integrating heterogeneous findings, this work provides an evidence-based framework for addressing this underrecognized source of dietary plastic exposure.

## 2 Methods

This study is a systematic review conducted in accordance with the PRISMA checklist. To minimize bias, all processes including article search, application of inclusion and exclusion criteria, and data extraction were independently carried out by two different authors.

### 2.1 Search strategy

The literature search in English was conducted up to 30 December 2025. No time restriction was applied in this systematic review. The databases selected for the search included PubMed, ScienceDirect, Web of Science, and Scopus. The search strategy used the following keywords: ("Microplastic" OR "micro-plastic" OR "nanoplastic" OR "nano-plastic") AND ("tea bag" OR ("teabag" OR "pyramid tea bag" OR "tea sachet"). In total, 118 articles were retrieved. Initially, the titles and abstracts were screened, and studies not meeting the inclusion criteria were excluded. Subsequently, the full texts were thoroughly reviewed by three authors (A.A, M.A.S, and M.E.S) to extract relevant information.

### 2.2 Inclusion and exclusion criteria

The three reviewers (A.A, M.A.S, and M.E.S) independently searched the databases using the selected keywords. Studies involving cell culture, soil/or water studies, in-vitro, in-vivo or animal models, other types of contamination, review papers, book chapters, tea leaves, and laboratory model systems were excluded. The inclusion criteria focused solely on original research articles examining MNPs contamination in tea bags. All studies meeting these criteria were included in the review.

### 2.3 Data extraction

The relevant data summarized in Table 1, Table 2 were extracted by two authors (A.A and M.A.S). Any disagreements at any stage were resolved through consultation with the corresponding author. The full texts of all included articles were accessible.

**Table 1:** Microplastics (MPs) in tea bags.

Ref.	Main findings	Risk/EDI/Health	Brewing time	Brewing temperature	MP shape	Detection technique	MP size	MP amount	MP type	N	Tea bag type	Country
Ahmad et al. (2025)	Tea bags were found to contain the highest MP load (615.7 particles/tea bag) among 112 food and drink products, highlighting significant daily and annual human exposure and the need for monitoring and mitigation strategies.	Daily and annual MPs intake (15.06 particles/kg/day and 5496.45 particles/kg/year, respectively)	-	-	-	FTIR	251–500 μm	615.71 particles/tea bag	PP, PE, PC, PVC	112	Tea bags	Kingdom of Saudi Arabia
Yue et al. (2024)	Woven tea bags emit far fewer MPs than nonwoven ones, and three pre-washes with room-temperature water can remove 76–94% of micron-sized and 80–87% of submicron-sized particles	-	5 min	80 °C	Fragments	LC-μ-Raman, Py-GC/MS	Micron-sized (>2.7) and submicron sized (<2.7)	80 to 1288 particles-0 to 63.755 μg	PET, PP, NY6	3	Intact plastic tea bags	China
Güzel İzmirli and Gökkaya (2024)	MPs were detected in 12 of 15 cup-of-tea bag brands (153 particles) and teapot tea bag brands (105 particles), with cup-of-tea bags accounting for 59.3% of MP-positive samples	Daily MP exposure from tea consumption ranged from 5.28 to 17.58 particles/mL for males and 4.16–13.85 particles/mL for females, with cup-of-tea bags resulting in 21.12 and 16.65 particles/mL/day for males and females, respectively	3 and 15 min	95 °C	Fiber	ATR-FTIR	33.65 μm to 1680.20 μm, with an average size of 197.40 ±13.13 μm.	3 to 33 particles/tea bag, with an average of 5.77 ± 0.55 particles/tea bag. Contamination values varied from 1.79 to 19.64	PP, EVA, PET, NY6, PVA, PAN, PA	15	Tea bags	Türkiye
Yousefi et al. (2024)	This study assessed human exposure to MPs from tea bags, finding 425,234–686,496 MPs/bag (average 518,459 ± 136,440), predominantly 10–50 μm in size (79%) with CA, PE, NY, and PET as main constituents, highlighting contamination sources in production and packaging and the resulting human and environmental exposure risks	EDI of MPs was approximately 17,282 particles/kg/day for children consuming 100 mL of tea daily and 14,813 particles/kg/day for adults consuming 400 mL of tea daily.	5 min	95 °C	74.67% Fibers, 18.67% fragments and 6.67% films	SEM	0–50 μm	Average abundance of 518,459 particles/tea bag	CA, NY, PET, PE	30	Tea bags	Iran
Kashfi et al. (2023)	Tea bags release high levels of MPs (mostly 100–250 μm fibers of PE and NY) and phthalates (mainly DEHP and DiBP)	Daily consumption of 150 mL of tea by children and 250 mL by adults may result in ingestion of approximately 486 and 810 MPs, respectively	-	-	-	-	100–250 μm	412.32 particles/tea bag	PE, NY	30	Tea bag/Iran	Iran
							100–250 μm	147.28 particles/tea bag	PE, NY	15	Tea bag/German	
Hernandez et al. (2019)	NY and PET particles released from tea bags were found at levels far exceeding those in other foods, with acute toxicity tests revealing dose-dependent behavioral and developmental effects in invertebrates.	Dialyzed leachates from NY and PET tea bags caused ingestion of MPs, developmental abnormalities, and altered swimming behavior in *Daphnia magna*, revealing sublethal biological impacts of MNPs.	5 min	95 °C	Spherical	FTIR, XPS	1–150 μm	11.6 billion (particles/one cup)	NY, PET	4	Tea bag	Canada
Xu et al. (2021)	Among six tea brands, NIR hyperspectral imaging revealed PP in four, NY in one, and no plastics in one biodegradable sample	-	10 min	95 °C	Spherical	FTIR	0.5∼5000 μm	1.3 × 10^10^ particles/pack	PP	6	Empty tea bags	Ireland
Kim et al. (2022)	Damaged tea bags release increasing amounts of fibrous MNPs and organic leachates with greater damage, higher temperatures, and longer exposure times, confirming NY leaching into hot water.	-	5 min to 1 h	25 – 70 °C	Fibers	SEM	>1 μm in size	-	NY	6	Damaged tea bag	South Korea
Mei et al. (2022)	Up to 94% of commercial filter bags (PE, PET, PP, NY6) released mostly tiny MP fragments and some fibers (620–840 μm) after soaking, with woven NY6 bags showing the lowest release	-	1 – 30 min	70 to 90 °C	Fragment and fiber	Raman	620–840 μm	56 MPs	PE, PET, PP, NY6	3	Tea bag	China
Cella et al. (2022)	Normal use of PE and NY food contact materials, including tea bags, can release MNPs, with up to 1.13 mg NY6/tea bag, and temperature strongly affects their morphology and aggregation, complicating classification and risk assessment.	-	10 min 2 h	95 °C −20 °C	Aggregation	FTIR	>0.45 μm	0.60 ± 0.10 mg	NY6	3	Pore Size 0.2 μm Hot-Filtered	Italy
								0.91 ± 0.14 mg			Pore Size 0.4 μm Hot-Filtered	
								0.92 ± 0.08 mg			Pore Size 2 μm Hot-Filtered	
								0.84 ± 0.14 mg			Pore Size 0.2 μm Cold-Filtered	
								0.93 ± 0.03 mg			Pore Size 0.4 μm Cold-Filtered	
								1.03 ± 0.10 mg			Pore Size 2 μm Cold-Filtered	
Ouyang et al. (2022)	An integrated pretreatment strategy was developed for simultaneous separation and enrichment of MPs and primary aromatic amines from tea bag migration, enabling sensitive one-step analysis for food safety and environmental risk assessment.	-	1 h	60 °C	Linear, spherical, irregular	ATR-FTIR, Raman	>10 μm and >1 μm	6 and 9	NY, PP	3	NY tea bag	China/spiked sample
			6 h	60 °C								
			24 h	60 °C								
			1 h	80 °C								
			6 h	80 °C								
			24 h	80 °C								
			1 h	100 °C								
			6 h	100 °C								
			24 h	100 °C								
			1 h	60 °C								
			6 h	60 °C								
			24 h	60 °C								
			1 h	80 °C								
			6 h	80 °C								
			24 h	80 °C								
			1 h	100 °C								
			6 h	100 °C								
			24 h	100 °C								
			1 h	60 °C				6	PET	3	PET tea bag	
			6 h	60 °C								
			24 h	60 °C								
			1 h	80 °C								
			6 h	80 °C								
			24 h	80 °C								
			1 h	100 °C								
			6 h	100 °C								
			24 h	100 °C								
Tabakoglu et al. (2024)	MPs release from tea bags into hot beverages exceeds that from plastic and paper cups, and elevated liquid temperatures further increase MP and endocrine-disrupting chemical exposure, highlighting the need for further health impact studies.	-	1-10 min	70 °C	Fibers and some fragments.	SEM, LC-MS/MS, FTIR	100–500 μm	At 90 °C: an average of 51,786 ± 1602 particles/cm^3^	PP	20	Tea bags	Türkiye
				50 °C				45,891 ± 671 particles/cm^3^				
Nikolaevich and Nickolaevna (2021)	Pyramidal tea bags released an enormous number of 1–2 nm NPs hundreds of millions of times more than MPs while paper tea bags released none, highlighting a dramatic difference in NPs contamination.	-	5 min	95 °C	Irregular fragments	DLS via Zeta analyzer	-	0	-	-	Paper tea bags	Russia
		-	-	-	-	-	-	0.01% of the total particles	PP, PE, PET	-	Pyramidal/polymeric tea bags	
Malynka et al. (2023)	Eight Ukrainian pyramid tea bag brands (Sun Gardens, Lovare, Curtis, Lipton, Premiya, Sonnet, Loyd) were analyzed: pyramids are PET (samples 1–7) or PLA (sample 8), threads are PP, PET, or PLA; woven pyramids (samples 1–3) have uniform 48–54 μm fibers, nonwoven (samples 4–8) have irregular 12–18 μm fibers, threads are twisted ply yarns (staple or filament), and steeping at 95 °C for 5 min releases irregular MPs from both pyramids and threads.	-	5 min	95 °C	Irregularity of structure, fiber type	FTIR, Optical microscopy	10–50 μm	-	PET, PP, PLA	8	Pyramid tea bags	Ukraine
(Yaroslavov et al., 2025)	Common tea bags made of synthetic (NY, PP) or natural (cellulose) polymers release large numbers of MNPs during brewing. Released particles (200 nm-1 μm, ∼600 nm average, bimodal distribution) increase in concentration with higher temperature and longer brewing times.	No cytotoxicity on Caco-2 cells	60 min	20; 50 and 100 °C	-	IR, DLS, EPM, NTA, DSC, TG	200 nm to 1 μm	Synthetic: 14 × 10^9^ particles/L Cellulose: 170 × 10^9^ particles/L	NY, PP, Cellulose	8	Tea bags	Russia

**Abbreviations**; MPs: Microplastics, NPs: Nanoplastics, EDI: Estimated Daily Intake, MNPs: Micro-nanoplastics, PP: Polypropylene, PE: Polyethylene, PC: Polycarbonate, PVC: Polyvinylchloride, PET: Polyethylene terephthalate, NY6: Nylon 6, NY: Nylon, EVA: Ethylene vinyl acetate, PVA: Polyvinyl alcohol, PAN: Polyacrylonitrile, PA: Polyamide, CA: Cellulose acetate, PLA: Polylactic acid.

FTIR: Fourier transform infrared spectroscopy, LC-μ-Raman: Liquid chromatography-micro-Raman spectroscopy, Py-GC/MS: Pyrolysis-gas chromatography/mass spectrometry, ATR-FTIR: Attenuated total reflectance – Fourier-transform infrared spectroscopy, SEM: Scanning electron microscopy, XPS: X-ray photoelectron spectroscopy, NIR: Near-infrared spectroscopy, LC-MS/MS: Liquid chromatography/mass spectrometry, DLS: Dynamic light scattering, IR: Infrared spectroscopy, EPM: Electrophoretic mobility, NTA: Nanoparticle tracking analysis, DSC: Differential scanning calorimetry, TGA: Thermogravimetry analysis.

**Table 2:** Nanoplastics (NPs) in tea bags.

Refs.	Main findings	Risk/EDI/Health	Brewing time	Brewing temperature	NP shape	Detection technique	NP size	NP amount	NP type	N	Tea bag type	Country
Banaei et al. (2024)	NPs released from tea bags (NY, PP, cellulose) showed variable abundances and were taken up by human intestinal cells in a cell type- and composition-dependent manner.	After 24 h at 100 μg/mL, PP-NPs were strongly taken up by HT29-MTX cells, CL-NPs showed similar uptake in HT29 and HT29-MTX cells, while NY6-NPs were mainly internalized in Caco-2 cells	-	95 °C	Spherule	ATF-FTIR	PP; 136.7 nm and NY6; 138.4 nm	PP: 1.20 × 10^9^/mL and NY6: 8.18 × 10^6^/mL	PP, NY6, Cellulose	1	Empty biodegradable tea bags	Spain
Hernandez et al. (2019)	NY and PET particles released from tea bags were found at levels far exceeding those in other foods, with acute toxicity tests revealing dose-dependent behavioral and developmental effects in invertebrates.	Dialyzed leachates from nylon and PET tea bags caused ingestion of micro-sized particles, developmental abnormalities, and altered swimming behavior in *Daphnia magna*, revealing sublethal biological impacts of MNPs.	5 min	95 °C	Spherical	FTIR, XPS	(<100 nm in size)	3.1 billion	NY, PET	4	Tea bag	Canada
Cella et al. (2022)	Normal use of PE and NY food contact materials, including tea bags, can release MNPs, with up to 1.13 mg NY6/tea bag, and temperature strongly affects their morphology and aggregation, complicating classification and risk assessment.	-	10 min	95 °C	Aggregation	FTIR	>20 nm	1.13 mg	NY6	3	Tea bag Pore Size 0.02 μm Hot-Filtered	Italy
			2 h	−20 °C								
								0.71 ± 0.10 mg			Pore Size 0.02 μm Cold-Filtered	
Nikolaevich and Nickolaevna (2021)	Pyramidal tea bags released an enormous number of 1–2 nm NPs hundreds of millions of times more than MPs while paper tea bags released none, highlighting a dramatic difference in NPs contamination.	-	5 min	95 °C	Spherical	DLS via Zeta analyzer	-	0	-	-	Paper tea bags	Russia
							1.1 nm	7.9 × 10^18^	PP, PE, PET	-	Pyramidal/polymeric tea bags	

**Abbreviations**; MPs: Microplastics, NPs: Nanoplastics, EDI: Estimated Daily Intake, MNPs: Micro-nanoplastics, PP: Polypropylene, PE: Polyethylene, PET: Polyethylene terephthalate, NY6: Nylon 6, NY: Nylon.

FTIR: Fourier transform infrared spectroscopy, ATR-FTIR: Attenuated total reflectance – Fourier-transform infrared spectroscopy, XPS: X-ray photoelectron spectroscopy, DLS: Dynamic light scattering.

## 3 Results

### 3.1 Systematic review findings

**Fig. 2:**
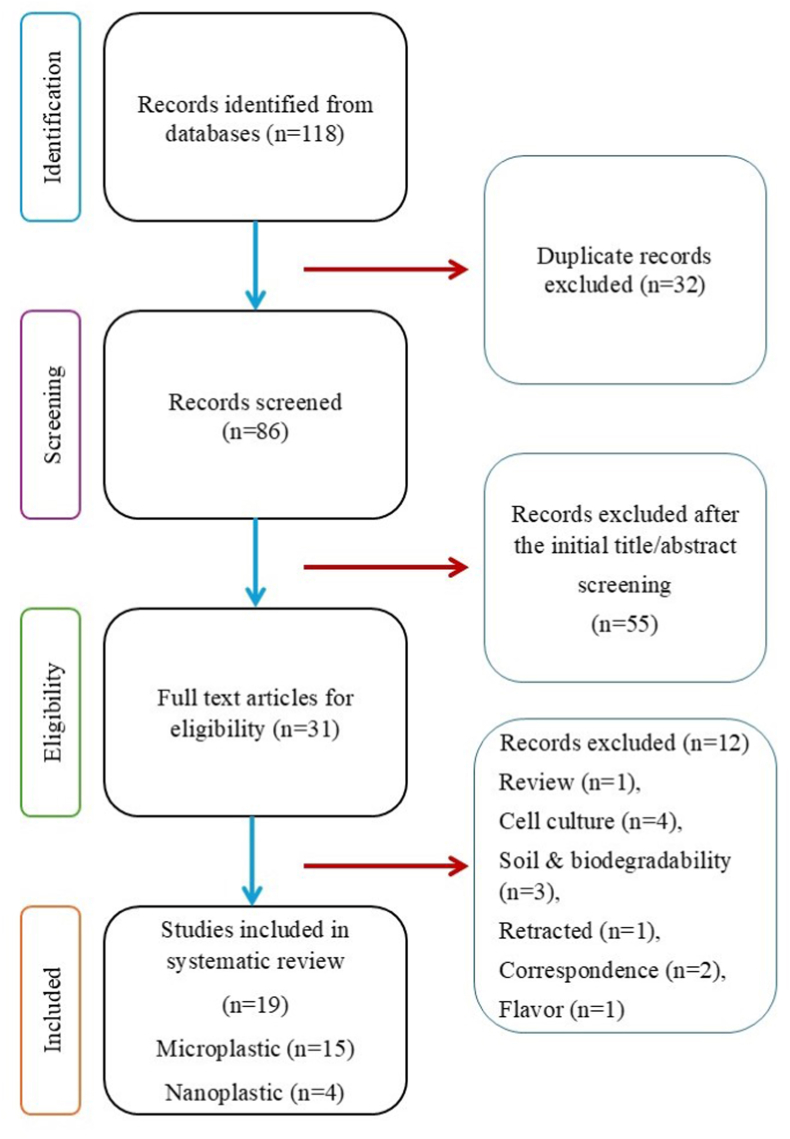
The standardized visual diagram of the study selection process in a systematic review, from initial identification of records to final inclusion of studies based on PRISMA guideline.

A total of 118 articles were identified through searches in PubMed, Scopus, Web of Science, and Science Direct. Of these, 32 studies were excluded as duplicates. The titles and abstracts of the remaining records (n = 86) were screened, leading to the exclusion of 55 articles. Subsequently, the full texts of 31 articles were evaluated by two reviewers, leading to the exclusion of 12 articles, due to reliance on in-vitro or animal models, different contamination types, review/correspondence papers, book chapters, tea leaves, or laboratory model systems. Ultimately, 19 studies met the eligibility criteria; 15 studies were explored microplastics (MPs) and 4 studies were explored nanoplastics (NPs). The flowchart of the database search process is illustrated in Fig. 2.

### 3.2 The descriptive results of screened articles

At this stage, 19 articles were included in the systematic review. Details such as country of study, type of tea bag packaging, sample size (N), type and quantity of MNPs, particle size, detection methods, particle shape, experimental temperature and time, risk/EDI/health assessments, notes, and references are presented in Table 1, Table 2

**Fig. 3:**
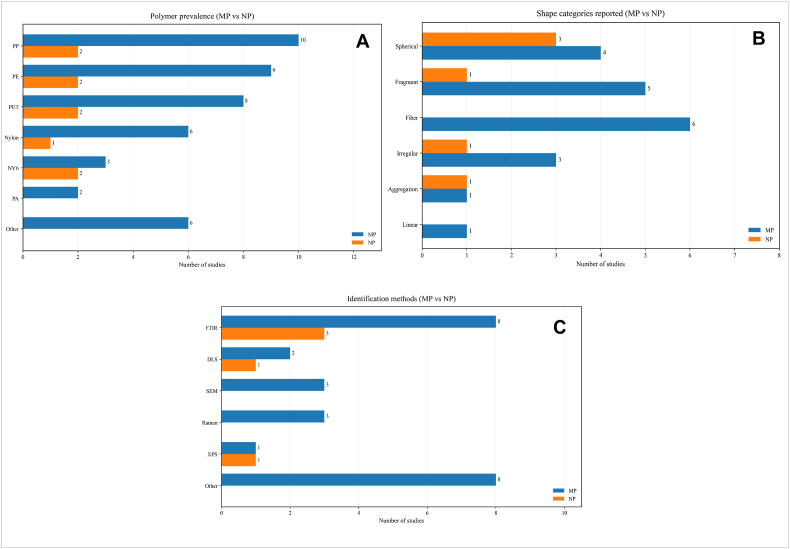
**(A)** Grouped horizontal bars showing the frequency with which individual polymer types were reported in the MP and NP datasets. Polymers identified in only one study (EVA, CA, PC, PAN, PLA, and PVA) were combined under Other for clarity, **(B)** Grouped horizontal bars showing the particle shape categories reported in the MP and NP datasets, **(C)** Grouped horizontal bars showing the analytical methods reported for MP and NP identification in tea-related studies (study-level counts). For clarity, single-occurrence methods were grouped as “Other”, including IR spectroscopy, NTA, EPM, DSC, TG, Py-GC/MS, LC-MS/MS, and optical microscopy.

The results of our systematic review, presented in Fig. 3A, B, and C, respectively illustrate the frequency of polymer types, polymer shapes, and the analytical methods used to identify MNPs. As shown in Fig. 3A, the most frequently investigated and reported polymer types were PP, PE, PET, Nylon, and Nylon-6. Regarding particle shape, the most commonly observed and reported categories were fibers, fragments, and sphericals (Fig. 3B). Among the analytical techniques used for MNPs characterization, FTIR, Raman spectroscopy, SEM, DLS, and XPS were the most widely applied (Fig. 3C).

**Fig. 4:**
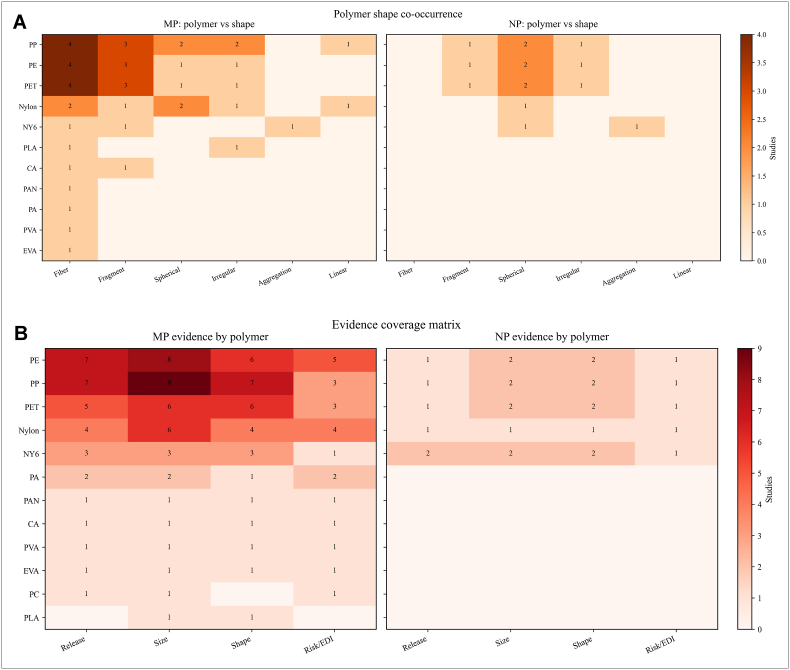
**(A)** Heat maps showing the co-occurrence of polymer type and particle shape in the MP and NP datasets, expressed as the number of studies reporting each combination, **(B)** Heat maps showing the number of studies that reported release, size, shape, and risk/estimated daily intake information for each polymer in the MP and NP datasets.

Fig. 4A displays a heatmap analysis of the correlation between polymer shape and polymer type, separately for MPs and NPs. Fig. 4B illustrates the relationship between the studied polymers and the parameters of release, size, shape, and EDI/risk. For MPs, the most frequently reported polymer shapes were fibers and fragments, associated with PP, PE, PET, and Nylon. In contrast, for NPs, the most commonly reported polymer shape was spherical (Fig. 4A). Concerning the investigated properties (Fig. 4B), size, release, and shape, along with EDI/risk, respectively, showed the highest frequency for MPs. However, for NPs, size and shape were the primary focus of investigation.

**Fig. 5:**
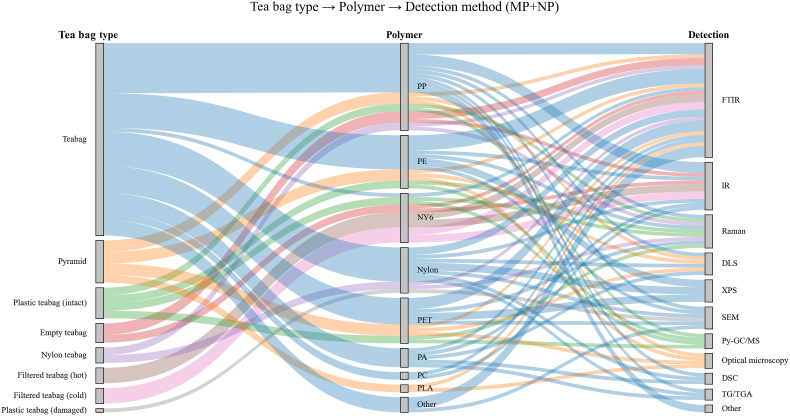
Sankey diagram illustrating the study-level relationships among tea bag type, reported polymer type, and analytical detection method across the combined MP and NP datasets. Polymer types and analytical methods reported only once were grouped under Other (polymers: EVA, CA, PAN, and PVA; methods: DSC and TG/TGA). ∗For clarity, PET teabag and nylon teabag entries were grouped under the general teabag category.

A Sankey diagram (Fig. 5), illustrates the relationship between the most frequently studied types of tea bags, the analyzed polymers, and the analytical techniques used. In this regard, for commonly consumed tea bags, PP was the most frequently reported polymer, and FTIR was the most widely used analytical method.

As an overall conclusion from the reviewed studies, polymers such as PP, PE, PET, Nylon, and Nylon-6 have garnered the most attention regarding MNPs release. Additionally, most studies have concentrated on MPs rather than NPs.

## 4 Discussion

### 4.1 Interpretation of findings

One of the most unexpected everyday sources of human exposure to microplastics and nanoplastics is tea bags made from plastic materials. When a typical tea bag (often nylon, polypropylene, or PET) is steeped in hot water, billions of tiny particles can be released into the drink frequently reaching over a billion particles per cup. This release is mainly triggered by the high temperature and hydrolytic breakdown of the polymer structure during brewing, which degrades the polymer structure and causes fragmentation. To examine this issue more clearly, the discussion is divided into two main parts: first, the characteristics, quantities, and factors affecting the release of microplastics from tea bags; and second, the release of nanoplastics, their different behavior, their combined effects on human health (such as cellular uptake, potential translocation in the body, and long-term low-level risks), and the important role of brewing time and water temperature in determining how many particles of both size ranges are emitted. This division helps highlight size-specific patterns while emphasizing the need for better tea bag materials and greater consumer awareness to reduce exposure from such a common daily habit.

**Microplastics (MPs):** The release of MPs from tea bags was studied in multiple countries worldwide, as detailed in Table 1. China was related to the highest number of studies assaying MPs (n = 3). In addition, 2 studies were conducted in Turkey, 2 in Iran, and 1 study each in Canada, Saudi Arabia, Ireland, South Korea, Italy, Russia, and Ukraine. The studies employed various analytical techniques for identification and quantification, with Fourier Transform Infrared Spectroscopy (FTIR) being the most commonly used method (Xu et al., 2021; Güzel İzmirli and Gökkaya, 2024; Ahmad et al., 2025), Raman spectroscopy (Mei et al., 2022; Ouyang et al., 2022; Yue et al., 2024), Scanning Electron Microscopy (SEM) (Kim et al., 2022; Tabakoglu et al., 2024), Pyrolysis-Gas Chromatography/Mass Spectrometry (Py-GC/MS) (Yue et al., 2024), and Dynamic Light Scattering (DLS) (Nikolaevich and Nickolaevna, 2021).

The most common shapes of MPs were fragments and fibers and the main polymer types were polyethylene (PE), polypropylene (PP), polyethylene terephthalate (PET), and nylon (polyamide). The size of MPs ranged from 1 μm (Yaroslavov et al., 2025) to 5000 μm (Xu et al., 2021) depending on the studies condition.

A study conducted in China (Yue et al., 2024) showed that woven plastic tea bags brewed at 80 °C for 5 min released between 80 and 1288 particles with size of more than 2.7 μm, substantially less than non-woven counterparts. When the bags were rinsed three times with water (at room temperature), the amount of microplastics was reduced by 76–94%. In another study of Tabakoglu et al., (2024) demonstrated that increasing the brewing temperature from 50 to 90 °C significantly increased MPs release from 45,891 to 51,786 particles/cm^3^. The study by Ouyang et al., (2022) found that increasing the temperature from 60 to 100 °C over 24 h increased the concentration of released MPs. It also confirmed that longer contact times (1 to 24 h) at a constant temperature resulted in higher leaching of MPs. The effect of temperature (25-75 °C) and brewing time (5 min to 1 h) on the release of microplastics was shown by Kim et al., (2022) that the highest amount of microplastics occurred at a temperature of 75 °C and a duration of 60 min. The number of MPs released from Iranian tea bags averaged 412.32 particles per bag, while the amount reported for German brands was 147.28 particles (Kashfi et al., 2023).

**Nanoplastics (NPs):** The release of NPs from tea bags was studied in four different countries (Spain, Canada, Italy, and Russia) (Table 2). These studies used various identification techniques; two studies used FTIR (Cella et al., 2022; Banaei et al., 2024), one study used DLS (Nikolaevich and Nickolaevna, 2021), and one study used both FTIR and X-ray Photoelectron Spectroscopy (XPS) (Hernandez et al., 2019). The studies examined various tea bag types, including biodegradable, polymeric bags (e.g., nylon and polypropylene), and paper bags. Three of the studies reported the results by number of particles and one provides the total mass (in mg) of plastic. The most common shapes identified were spherical.

Temperature and time of brewing are major factors influencing the release and particle behavior. Four studies evaluated the standard tea brewing temperature (∼95 °C). This temperature significantly increases the release of NPs owing to degradation of polymers (Hernandez et al., 2019). A study carried out in Italy found that the release nylon-6 was more than 60% (1.13 mg at 95 °C for 10 min compared to 0.71 mg at −20 °C for 2h) with mean size of >20 nm at the high temperature (Cella et al., 2022). High temperature not only increased the release rate but also changed particle morphology. At 95 °C, nanoparticles tend to aggregate and form irregular structures, whereas at lower temperatures the particles remain more dispersed. A study conducted in Canada, brewing tea at 95 °C for 5 min, reported the release of 11.6 billion microplastics (1–150 μm) and 3.1 billion nanoplastics (<100 nm) per nylon and PET tea bags (Hernandez et al., 2019). In 2021, Nickolaevna et al. (Nikolaevich and Nickolaevna, 2021) studied tea bags made of paper and polymeric materials at brewing of 95 °C for 5 min. They reported the release of up to 7.9 × 10^18^ nanoparticles in polymeric/pyramidal bags with mean size of 1.1 nm. In contrast, paper tea bags showed no measurable release of nanoplastics. A key difference between the samples is type of tea bag material, brewing parameters (temperature and agitation), and experimental conditions (sample size, instrumental sensitivity, and identification techniques) (Chen et al., 2023).

## 5 Limitations and strengths of the study

### 5.1 Strengths of the study

This systematic review offers the first comprehensive global synthesis of MNPs release from tea bags, incorporating evidence from diverse geographical regions and tea bag materials (including nylon, PET, polypropylene, PLA, and supposedly “paper” bags with plastic components). By integrating multidisciplinary data on release mechanisms, particle characteristics, quantification techniques, exposure estimates, and both human health and environmental implications, it provides a holistic overview of an everyday yet largely overlooked dietary exposure route that affects billions of tea consumers worldwide. The review critically evaluates methodological inconsistencies across studies, highlights the superiority of certain analytical approaches, and underscores the alarming scale of MNP ingestion (often billions of particles per cup) compared to other food sources. Its emphasis on additive leaching, nanoplastic bioavailability, and the misleading marketing of some “plastic-free” products adds significant value for public health awareness and regulatory discourse, making it a timely and policy-relevant contribution that bridges gaps between food safety, toxicology, and environmental science.

### 5.2 Limitations of the study

The review is constrained by considerable heterogeneity among primary studies, including variations in brewing conditions (temperature, duration, intact vs. cut bags, presence of tea leaves), analytical methods, and particle size thresholds, which prevent direct comparisons and formal meta-analysis. Most included studies fail to report central tendency measures (mean values) or measures of variability (standard deviation, confidence intervals, or raw data), further preventing quantitative synthesis and reducing the precision of overall exposure estimates. The field remains nascent, with a limited number of high-quality primary studies particularly for biodegradable or cellulose-based materials and potential publication bias toward high-releasing plastic tea bags. Technical challenges in detecting and quantifying nanoplastics (<1 μm), the most bioavailable fraction, lead to systematic underestimation. Moreover, nearly all evidence derives from controlled laboratory simulations rather than real consumer practices (e.g., repeated use, addition of additives/sugar), limiting ecological validity. Direct evidence of long-term human health consequences and environmental fate of discarded tea bags or released MNPs remains scarce, and possible language or regional bias may have excluded relevant non-English publications from major tea-consuming countries. These limitations collectively highlight the need for greater standardization and more robust primary research in the future.

## 6 Future research directions

Future research on micro/nanoplastics release from tea bags should highlight the development and validation of standardized experimental protocols for brewing conditions and particle quantification, with particular emphasis on reliable detection of nanoplastics below 1 μm. Studies need to shift toward more realistic consumer scenarios, including repeated bag use, addition of additives, sugar, or lemon, and variations in water quality and composition. As primary studies begin routinely reporting mean values, standard deviations, or raw data, quantitative meta-analyses will become feasible and should be conducted to provide more precise exposure estimates. Greater attention must be given to so-called “plastic-free” and biodegradable tea bags (e.g., PLA and cellulose-based) to determine their actual MNP release and chemical leaching under real-world conditions. Long-term human health impacts should be also investigated through cohort studies in high-tea-consuming populations and biomonitoring of plastic-associated chemicals in blood, urine, and tissues. Simultaneously, the environmental fate of discarded tea bags and released MNPs in wastewater, soil, and aquatic systems requires systematic evaluation. Finally, practical, low-cost mitigation strategies such as pre-rinsing tea bags, cold brewing, or switching to loose-leaf tea should be tested for effectiveness and promoted through public health guidance and regulatory measures.

## 7 Conclusion

This systematic review focused on detection and quantifying MNPs release and confirmed that conventional tea bags are a significant source of micro- and nanoplastics (MNPs) release into beverages during the brewing process. Studies included in this review consistently demonstrate that plastic-based tea bags, primarily composed of nylon, polyethylene terephthalate (PET), and polypropylene (PP), release substantial numbers of particles, often exceeding billions per cup, when exposed to hot water. This release occurs even in some products marketed as biodegradable or paper-based, suggesting widespread use of plastic components. The primary mechanism identified for this release is the thermal degradation and hydrolysis of plastic materials at high brewing temperatures and brewing long-time.

Further research is needed to standardize MNPs measurement methodologies, conduct long-term human exposure studies, assess the effects of real-world exposure mixtures, investigate the potential biological implications, and evaluate human health risks comprehensively.

To mitigate this issue, key steps should include the phasing out of plastic components in tea bags that lead to high particle release, the implementation of clear and accurate labeling regarding material composition, and the promotion of genuinely plastic-free alternatives like loose-leaf tea. Reducing reliance on single-use, plastic-containing tea bags through industry innovation and informed consumer choices is essential for minimizing both dietary MNPs exposure and environmental pollution.

## Author contributions statement

All authors written and reviewed the manuscript.

## Declaration of competing interest

The authors declare that they have no known competing financial interests or personal relationships that could have appeared to influence the work reported in this paper.
